# Application of distributed lag models and spatial analysis for comparing the performance of the COVID-19 control decisions in European countries

**DOI:** 10.1038/s41598-023-44830-z

**Published:** 2023-10-14

**Authors:** Ali Hadianfar, Sedigheh Rastaghi, Hamed Tabesh, Azadeh Saki

**Affiliations:** 1 Department of Epidemiology and Biostatistics, School of Health, Mashhad University of Medical Sciences, Mashhad, Iran; 2 Department of Medical Informatics, School of Medicine, Mashhad University of Medical Sciences, Mashhad, Iran

**Keywords:** Health care economics, Health policy, Statistics

## Abstract

Over the past three years, the COVID-19 outbreak has become a major worldwide problem, affecting the health systems and economies of countries. The mean delays, the expected time to observe the average effect of the number of new cases on the number of deaths, are gold times for decision-making regarding disease control and treatment facilities to reduce the fatality rate. The interest of the present study is estimating the mean delays and adjusted fatality rates of COVID-19 with the new application of Distributed Lag Models (DLM) and their spatial distributions. The daily cases and deaths data of COVID-19 for 39 European countries was obtained from two sources; the "European Centre for Disease Prevention and Control" and the "Our World in Data" database. The mean delay and the Adjusted Fatality Rate (AFR) for each country at three-time intervals; the first and subsequent peaks before and after vaccination were estimated by the Distributed Lag Models. The spatial analysis was applied to find the spatial correlation of the mean delays and adjusted fatality rates among European countries. In the three-time intervals, the first and the subsequent peaks before vaccination, and after vaccination, the median and interquartile range of the mean delays; and AFRs were: 1.1 (0.4, 3.2); 0.024 (0.016, 0.044), 9.2 (6.2, 12.40); 0.013 (0.005, 0.020) and 7.3 (4.4, 11.0); 0.001 (0.001, 0.005), respectively. In the subsequent peaks before vaccination, the mean delays considerably increased, and the AFRs decreased for most European countries. After vaccination, the AFRs decreased considerably. Except for the first peak, the spatial correlations of AFRs were not significant among neighboring countries. Consecutive outcomes will occur with delays in outbreaks of infectious disease. Also, the fatality rates for these outcomes should be adjusted on delays. Estimating the mean delays and adjusted fatality rates by Distributed lag Models and the spatial distributions of theme in outbreaks showed that prevention and medical policies after the first peak as well as vaccination were effective to reduce the fatality rate of COVID-19, but these effects were different between countries. These results recommended policymakers and governments assign prevention and medical resources more effectively.

## Introduction

The new coronavirus (COVID-19) outbreak began in Wuhan, China, towards the end of December of 2019 and was declared a global pandemic on March of the following year^1^. Over the past two years, this virus has become a global problem, harming healthcare systems and economies in various countries. In Europe, when it was first discovered in the north of Italy and Spain, it quickly spread across the continent. On July 07, 2022, the WHO estimated that 206,490,180 cases of Coronavirus infection and 1,858,450 fatalities had been reported in Europe. As a result of the high incidence of this disease, Europe has become the second deadliest continent followed by North America ^2,3^.

Detection of COVID-19 outbreak is sometimes postponed many weeks, and so making early decisions about required interventions is a challenge. A great deal of research has been done against delaying the diagnosis of infectious diseases^4–7^. Research shows that late diagnosis of COVID-19 could increase infections and have been linked to many deaths^4,8,9^. Therefore, the important indices for predicting and managing COVID-19 medical resources in the community are the mean delay, the expected time to observe the average effect of a unit change in the number of new cases on the number of deaths, and the Adjusted Fatality Rate (AFR)^10^.

The reported mean delays between new cases and deaths of COVID-19, are different among countries^11^. A systematic review shows that the incubation period of COVID-19 is about six to seven days. Screening tests can diagnose patients at the onset of disease and so increase the delay time between diagnosis, hospitalization, and death^12^.

Previous studies have shown a distinct delay between the peak of daily COVID-19 infections and the peak of daily deaths. Two major components of health, early diagnosis of infection and the level of treatment facilities, can impact this delay. A greater delay time is expected in countries with superior health systems^13,14^ and so, this delay time was varied among countries with different level of healthcare^15,16^.

The number of deaths at each day is affected by the number of new cases during previous days. Also, these effects are not homogeneous and considerably different according to time of diagnosis. So, ordinary fatality rate without adjusting on the delay times between diagnosis and death is biased^4,8,9,17^.

According to previous studies^8,11^, when a new wave of COVID-19 cases appears, hospitalizations and deaths increase, but the time and amount of these increases vary according to population characteristics of nations. Research in England has shown that it takes between two and eight weeks for a person to develop symptoms and die from COVID-19^18^. The median time between the onset of symptoms and death is more than 14 days^18,19^. In addition, the median time between admission and mortality in the intensive care unit was between 7 and 12.5 days^18,20^. As a result, the impact of new cases on future COVID-19 deaths must be considered.

In present study the Distributed Lag Model (DLM) was used for predicting future deaths according to the daily number of new cases in previous days. Also, this model could be estimating both important indices: the mean delay and AFR of COVID-19 in socio-economic and epidemiological studies^21,22^. So, the interest of this study is to estimate the mean delay and AFR of COVID-19 among European countries and their spatial distributions with new application of DLMs that anticipate the delay effect of the number of COVID-19 cases on fatality rate.

## Methods

### Data

The daily cases and deaths data of COVID-19 for 39 European countries was obtained from two sources; the "European Centre for Disease Prevention and Control" and "Our World in Data" database^23^ both data sets freely publishing online, the daily number of new COVID-19 cases and deaths by countries. We used data from January 1, 2020, to May 1, 2022 since some nations began vaccination campaigns against COVID -19 in 2021. The first peak of COVID-19 in European countries was from January 1, to August 31, of 2020 and between September 1, 2020 and April 30, 2021 these countries experience two or more peaks, according to a time-series analysis. These time interval were chosen to compare the performance of health care systems of the European countries at the first and the subsequent peaks of the COVID-19. Also, to assessing the effect of vaccination on fatality rates of CIVID-19 of those countries data between May 1, 2021, and May 1, 2022, was used. It was assumed that the outbreak of the new COVID-19 variants, and vaccination in European countries was approximately simultaneous.

### Data analysis

#### Distributed Lag Models (DLMs)

Research in environmental health and epidemiology have examined the possibility of delayed effects. A tool originally developed for economic modeling, Distributed Lag Models (DLMs), is commonly used to explain delayed effects. In environmental epidemiology these models have been used widely^24–26^. For example, in COVID-19 disease, death occurred with various delays after diagnosis. So adjusting the fatality rate is necessary regarding the delayed effects of COVID-19 new cases in the preceding days that can be estimated by the DLMs^8,9^ Also, the mean of delays is very different between countries that may due to the health disparities.

DLMs have two types: finite and infinite lags; infinite distributed lags allow the current value of the dependent variable to be influenced by values of the independent variable that occurred infinitely long ago; but beyond some lag length, the effects taper off toward zero. On the other hand, finite distributed lags allow for the independent variable at a particular time to influence the dependent variable for only a constrained number of lags^27,28^. In the present study we used finite DLM, and two most common type of transformed coefficient models; Koyck-DLM and polynomial-DLM.

In a DLMs, the effect of an exploratory variable *X* on dependent variable *Y* appear with a delay and is distributed over time. This study considers a daily number of new reported COVID-19 cases and deaths as explanatory and dependent variables, respectively. Let ${Y}_{t}$ be the number of COVID-19 deaths at day $t;t=1,\dots T$ and ${X}_{t-l}$ be the number of COVID-19cases at day $-l;l=\mathrm{0,1},\dots$ , the infinite DLM can be written as follow:

1 $${Y}_{t}=\alpha +\sum_{l=0}^{\infty }{\beta }_{l}{X}_{t-l}+{\epsilon }_{t}$$

where E(${\epsilon }_{t}$) = 0 and var (${\epsilon }_{t}$) = c, α is intercept, β-parameters are the effect of the changes in ${X}_{t}$ on the expected value of ${\mathrm{Y}}_{\mathrm{t}}$. Estimation of an infinite number of $\upbeta$ coefficients is impossible, and it is usually assumed that ${\mathrm{lim}}_{\mathrm{i}\to \infty }{\upbeta }_{\mathrm{i}}=0$ and $\sum_{\mathrm{i}=0}^{\infty }{\upbeta }_{\mathrm{i}}=\upbeta <\infty$. Assuming that the changes in X_t_ do not have much effect after L number of days, the proposed model reduces to a finite distributed lag model as follows:

2 $${\mathrm{Y}}_{\mathrm{t}}=\mathrm{\alpha }+\sum_{\mathrm{l}=0}^{\mathrm{L}}{\upbeta }_{\mathrm{l}}{\mathrm{X}}_{\mathrm{t}-\mathrm{l}}+{\upepsilon }_{\mathrm{t}}$$

Bounded summing of finite length L yields a finite linear DLM of order L, which is the only solution. The n-1 [${\mathrm{y}}_{\mathrm{t}}.{\mathrm{x}}_{\mathrm{t}}$] pairs are applied to evaluate model parameters. These distributed lag models incorporate several parameters that should not be overlooked. A conservative assumption is that the lagged variable coefficients are not all independent, but rather functionally connected. In this model, we consider maximum lag equal 14 based on countries’ reports and previous studies^10,11^; then, according to the Akaike information criterion (AIC), the ideal number of lag was selected for each country. In this situation, the number of COVID-19 deaths at a given time t may be explained in the past number of new cases ${\mathrm{X}}_{\mathrm{t}-\mathrm{l}}$ with $\mathrm{l}$ as the lag, representing the period elapsed between the independent variable and the response. So our model is as follows:

3 $${\mathrm{number~of~deaths}}_{\mathrm{t}}=\mathrm{\alpha }+\sum_{\mathrm{l}=0}^{\mathrm{L}}{\upbeta }_{\mathrm{l}}{*\mathrm{number~ of~cases}}_{\mathrm{t}-\mathrm{l}}+{\upepsilon }_{\mathrm{t}}$$

Through this model, the mean delay and long-run effect (AFR) can be estimated as follows:

4 $$\mathrm{mean~delay}=\frac{\sum_{\mathrm{l}=0}^{\mathrm{L}}{\upbeta }_{\mathrm{l}}*\mathrm{l}}{\sum_{\mathrm{l}=0}^{\mathrm{L}}{\upbeta }_{\mathrm{l}}}$$

5 $$\mathrm{long}-\mathrm{run\; effect }(\mathrm{AFR})=\sum_{\mathrm{j}=0}^{\mathrm{L}}{\upbeta }_{\mathrm{j}}={\upbeta }_{0}+{\upbeta }_{1}+\dots {\upbeta }_{\mathrm{L}}$$

The mean delay is the weighted average of lags that shows how long it takes the average effect of change in the number of cases on the number of deaths. The long-run effect is the overall effect of daily new cases on death that equals to the AFR according delays.

Koyck-DLM model is an infinite DLM. Due to the unrestricted lags, the number of coefficients in the above model is infinite, making it necessary to impose restrictions on their structure. The appropriate solution for this task is to use the Koyck transformation, which defines the structure of the coefficients as follows:

6 $${\upbeta }_{\mathrm{l}}={\upbeta }_{0}{\uplambda }^{\mathrm{l}} 0\le\uplambda \le 1$$

By replacing this equation in (1) the Koyck -DLM is as follow:

7 $${\mathrm{number \;of \;death}}_{\mathrm{t}}=\mathrm{\alpha }+\sum_{\mathrm{l}=0}^{\infty }{\upbeta }_{0}{\uplambda }^{\mathrm{l}}{*\mathrm{number~of~cases}}_{\mathrm{t}-\mathrm{l}}+{\upepsilon }_{\mathrm{t}}$$

For this model, the mean delay and long-run effect (AFR) are estimated by:

8 $$\mathrm{mean \;delay}=\frac{\sum_{\mathrm{l}=0}^{\infty }{\upbeta }_{\mathrm{l}}*\mathrm{l}}{\sum_{\mathrm{l}=0}^{\infty }{\upbeta }_{\mathrm{l}}}=\frac{\uplambda }{1-\uplambda }$$

9 $$\mathrm{long}-\mathrm{run~ effect }(\mathrm{AFR})=\sum_{\mathrm{j}=0}^{\mathrm{L}}{\upbeta }_{\mathrm{j}}=\frac{{\upbeta }_{0}}{1-\uplambda }$$

Polynomial-DLM is a finite DLM model, where ${\upbeta }_{\mathrm{l}}\mathrm{s}$ are Polynomial equations of degree r as follow:

10 $${\upbeta }_{\mathrm{l}}={\mathrm{\alpha }}_{0}+{\mathrm{\alpha }}_{1}\mathrm{l}+{\mathrm{\alpha }}_{2}{\mathrm{l}}^{2}+\dots +{\mathrm{\alpha }}_{\mathrm{r}}{\mathrm{l}}^{\mathrm{r}}$$

By replacing this equation in (2) for r = 2 the Polynomial-DLM is as follow:

11 $${\mathrm{number \;of \;death}}_{\mathrm{t}}=\mathrm{\alpha }+\sum_{\mathrm{l}=0}^{\mathrm{L}}({\mathrm{\alpha }}_{0}+{\mathrm{\alpha }}_{1}\mathrm{l}+{\mathrm{\alpha }}_{2}{\mathrm{l}}^{2}){*\mathrm{number~ of~ cases}}_{\mathrm{t}-\mathrm{l}}+{\upepsilon }_{\mathrm{t}}$$

For this model, the mean delay and long-run effect (AFR) are estimated by:

12 $$\mathrm{mean \;delay}=\frac{\sum_{\mathrm{l}=0}^{\mathrm{L}}{\upbeta }_{\mathrm{l}}*\mathrm{l}}{\sum_{\mathrm{l}=0}^{\mathrm{L}}{\upbeta }_{\mathrm{l}}}= \frac{\mathrm{L}}{2}\left({\mathrm{\alpha }}_{0}+{\mathrm{\alpha }}_{1}\frac{2\mathrm{L}+1}{3}+{\mathrm{\alpha }}_{2}\frac{\mathrm{L}\left(\mathrm{L}+1\right)}{2}\right)/\left({\mathrm{\alpha }}_{0}+{\mathrm{\alpha }}_{1}\frac{\mathrm{L}}{2}+{\mathrm{\alpha }}_{2}\frac{\mathrm{L}\left(2\mathrm{L}+1\right)}{6}\right)$$

13 $$\mathrm{long}-\mathrm{run \;effect }(\mathrm{AFR})=\sum_{\mathrm{j}=0}^{\mathrm{L}}{\upbeta }_{\mathrm{j}}= {\mathrm{\alpha }}_{0}(\mathrm{L}+1)+{\mathrm{\alpha }}_{1}\frac{\mathrm{L}(\mathrm{L}+1)}{2}+{\mathrm{\alpha }}_{2}\frac{\mathrm{L}\left(\mathrm{L}+1\right)(2\mathrm{L}+1)}{6}$$

Due to our data structure the predictive powers of these three models were compared by Median Absolute Percentage Error (MdAPE). The MdPAE is the median of the Absolute Percentage Errors (APE_t_) that is defined as:

14 $${APE}_{t}=\left|\frac{\left({\widehat{y}}_{t}-{y}_{t}\right)}{{y}_{t}}\right|$$

The best model is a model with the smaller value of the MdAPE.

#### Spatial analysis of the mean delays and AFRs

Moran's bivariate spatial correlation was used to find the effect of outbreaks in neighboring countries on the mean delays and AFRs among European countries. As the number of death was significantly correlated with the number of new cases, we used Moran's bivariate spatial correlation to examine the correlation between the mean delay and AFR with the number of new cases among neighboring countries.

Also, spatial maps were drowning to present the spatial distribution of the mean delays and AFRs in Europe.

R4.2.2 was used for statistical computation. We employ the “dLagM” and “tmap” package for fitting DLMs, and spatial mapping, respectively.

## Ethical approval and consent to participate

In this study open-source data set was used. And the methodology was used for these data approved at ethics approval board of the Research of Mashhad University of Medical Sciences.

## Results

Results from the fitting the distributed lag model to data on the daily number of newly reported COVID-19 cases and deaths in 39 European countries revealed that there was heterogeneity among countries in respect of the values of the mean delay and AFR before vaccination in both the first and the subsequent peaks as well as after vaccination. The results of three models showed that for most countries the Koyck-DLM model was better than the two other models (supplementary file, S1). The results of the Koyck-DLM models are shown in Table 1. The results of these models indicated that the mean delay between the number of new cases and the daily death of COVID-19 among European countries in the first half of 2020 varied between 0 and 17.7 days. The lowest average delays were in the Cyprus, North Macedonia, and Latvia, with the mean delay at 0.0, 0.1, and 0.1 respectively. The highest average delays were found in Spain, Sweden, and Belgium and their respective delays were 17.7, 16.0 and 12.0 days. The average delay time between European countries during this period was 2.9 ± 4.2 days, which means that the impact of any decrease or increase in the number of new cases on the number of deaths appears after 2.9 days.

**Table 1:** The Mean Delay and Adjusted Fatality Rate (AFR) of COVID-19 Estimated by Koyck-DLMs among European countries from January 2020 to May 2022.

Country	1-Jan-2020 to 31-Aug-2020		1-Sep-2020 to 30-Apr-2021		1-May-2021 to 1-May 2022	
	Mean Delay	AFR	Mean Delay	AFR	Mean Delay	AFR
Albania	0.4	0.031	2.1	0.014	3.1	0.003
Austria	5.4	0.026	13.7	0.020	10.8	0.001
Azerbaijan	0.7	0.014	7.9	0.012	5.0	0.006
Belarus	4.1	0.005	3.8	0.003	4.5	0.001
Belgium	12.0	0.185	16.6	0.010	4.9	0.001
Bosnia & Herzegovina	0.7	0.023	11.0	0.023	11.2	0.023
Bulgaria	0.7	0.021	12.6	0.030	6.9	0.015
Croatia	0.7	0.006	6.2	0.019	7.3	0.006
Cyprus	0.0	0.012	0.5	0.003	6.9	0.001
Czech IA	3.1	0.010	11.6	0.011	23.5	0.002
Denmark	1.3	0.052	5.4	0.007	2.1	0.001
Estonia	0.2	0.024	11.5	0.007	0.7	0.001
Finland	0.7	0.048	0.6	0.005	7.3	0.003
France	3.4	0.111	14.3	0.004	10.8	0.001
Germany	9.0	0.024	9.1	0.018	11.0	0.001
Greece	0.9	0.011	8.4	0.026	7.8	0.001
Hungary	1.6	0.088	16.2	0.028	14.8	0.008
Ireland	0.4	0.066	10.2	0.013	1.7	0.000
Italy	1.9	0.014	8.0	0.020	10.7	0.002
Kosovo	0.4	0.044	8.6	0.014	5.9	0.005
Latvia	0.1	0.009	6.0	0.015	2.1	0.001
Lithuania	0.1	0.020	11.3	0.014	5.3	0.001
Moldova	0.3	0.019	7.2	0.002	4.8	0.005
Netherlands	3.2	0.106	2.2	0.003	4.4	0.000
North Macedonia	0.1	0.038	11.7	0.026	3.2	0.010
Norway	1.1	0.016	19.4	0.003	33.6	0.000
Poland	2.0	0.018	13.7	0.001	8.5	0.010
Portugal	2.8	0.040	11.0	0.023	3.3	0.001
Romania	1.4	0.029	12.4	0.003	7.8	0.004
Russia	2.8	0.016	6.8	0.014	15.0	0.031
Serbia	1.7	0.023	9.1	0.009	13.4	0.004
Slovakia	0.3	0.006	3.4	0.026	15.5	0.000
Slovenia	0.9	0.029	9.2	0.005	3.5	0.001
Spain	17.7	0.041	4.2	0.011	4.8	0.001
Sweden	16.0	0.020	14.9	0.006	9.2	0.001
Switzerland	8.0	0.060	11.6	0.018	3.0	0.000
Turkey	5.8	0.030	12.0	0.006	19.8	0.003
Ukraine	0.8	0.016	7.7	0.016	10.9	0.003
United Kingdom	0.7	0.239	13.2	0.033	14.8	0.001

Furthermore, results from the fitting of models to data from the second time interval showed that the mean delay had increased in all European countries. The overall mean delay was observed to be 9.4 ± 4.6 days, and it varied between 0.5 and 19.4 days. Also, in these six months, the lowest mean delay was seen in Cyprus, Finland, and Albania, with the average delay at 0.5, 0.6, and 2.1 days, respectively, and the highest delay was noted in Norway, Belgium, and Hungary at 19.4, 16.6, and 16.2 days, respectively.

In addition, there is a considerable variation among different countries regarding the long-run effect, which shows the AFR based on new cases identified in the previous days. Thus, in the first six months of 2020, the highest values of this index belonged to three countries, namely, England, Belgium and Italy. They had adjusted fatality rates of 0.24, 0.19 and 0.14 respectively. On the contrary, the lowest value was observed in Belarus, Slovakia, and Latvia with the AFR 0.005, 0.006 and 0.009 respectively. Furthermore, our findings indicate that this index decreased in most countries in the second time interval, indicating a decrease in fatality rates. For example, in the case of Britain and France, this index decreased to 0.03 and 0.004, respectively.

Table 2 shows the result of the models goodness of fits. As the MdAPEs of the Koyck-DLMs were smaller than the Finite-DLMs and the Polynomial-DLMs this model was the best for these data sets (these indices are reported in the supplementary file S1). The adjusted R-squared values are high in most countries, which indicates a suitable fit of the models for these data sets. Also, for cross validation, fourteen days’ forecast based on the Koyck-DLM model with their upper and lower 95% confidence limits were presented at supplementary file S2.

**Table 2:** Adjusted R-Squared (Adj. R^2^) and Median Absolute Percentage Error (MdAPE)MdAPE for the Koyck –DLMs fitted to European countries COVID-19 data from January 2020 to May 2022.

Country	1-Jan-2020 to 31-Aug-2020		1-Sep-2020 to 30-Apr-2021		1-May-2021 to 1-May 2022	
	Adj. R^2^	MdAPE	Adj. R^2^	MdAPE	Adj. R^2^	MdAPE
Albania	0.74	0.14	0.78	0.08	0.66	0.11
Austria	0.80	0.13	0.93	0.04	0.87	0.05
Azerbaijan	0.84	0.09	0.96	0.05	0.86	0.07
Belarus	0.79	0.09	0.80	0.03	0.70	0.03
Belgium	0.98	0.20	0.95	0.02	0.79	0.08
Bosnia & Herzegovina	0.59	0.36	0.66	0.12	0.61	0.21
Bulgaria	0.45	0.34	0.80	0.24	0.51	0.30
Croatia	0.22	0.33	0.89	0.07	0.89	0.06
Cyprus	0.20	0.04	0.20	0.37	0.26	0.31
Czech IA	0.63	0.18	0.93	0.02	0.94	0.03
Denmark	0.78	0.37	0.78	0.13	0.64	0.27
Estonia	0.48	0.12	0.62	0.28	0.32	0.31
Finland	0.66	0.19	0.30	0.29	0.74	0.21
France	0.69	0.45	0.10	0.37	0.69	0.22
Germany	0.87	0.36	0.66	0.20	0.79	0.09
Greece	0.46	0.36	0.85	0.04	0.82	0.05
Hungary	0.75	0.17	0.91	0.02	0.93	0.04
Ireland	0.87	0.49	0.88	0.06	0.51	0.16
Italy	0.96	0.25	0.82	0.03	0.87	0.05
Kosovo	0.77	0.22	0.53	0.20	0.79	0.12
Latvia	0.20	0.11	0.52	0.31	0.49	0.23
Lithuania	0.17	0.15	0.80	0.15	0.74	0.08
Moldova	0.55	0.40	0.78	0.03	0.73	0.12
Netherlands	0.83	0.37	0.51	0.18	0.62	0.15
North Macedonia	0.57	0.23	0.67	0.13	0.67	0.11
Norway	0.49	0.26	0.31	1.05	0.10	7.63
Poland	0.60	0.20	0.51	0.16	0.56	0.68
Portugal	0.85	0.24	0.98	0.05	0.83	0.08
Romania	0.82	0.17	0.60	0.08	0.80	0.09
Russia	0.87	0.18	0.88	0.03	0.99	0.00
Serbia	0.85	0.06	0.98	0.03	0.98	0.02
Slovakia	0.23	0.06	0.61	0.12	0.88	0.04
Slovenia	0.51	0.16	0.84	0.06	0.71	0.12
Spain	0.92	1.08	0.79	0.09	0.81	0.08
Sweden	0.96	0.04	0.91	0.05	0.89	0.07
Switzerland	0.94	0.23	0.95	0.06	0.69	0.11
Turkey	0.99	0.01	0.99	0.02	0.95	0.02
Ukraine	0.84	0.21	0.68	0.09	0.82	0.07
United Kingdom	0.99	0.03	0.99	0.04	0.94	0.02

**Figure 1:**
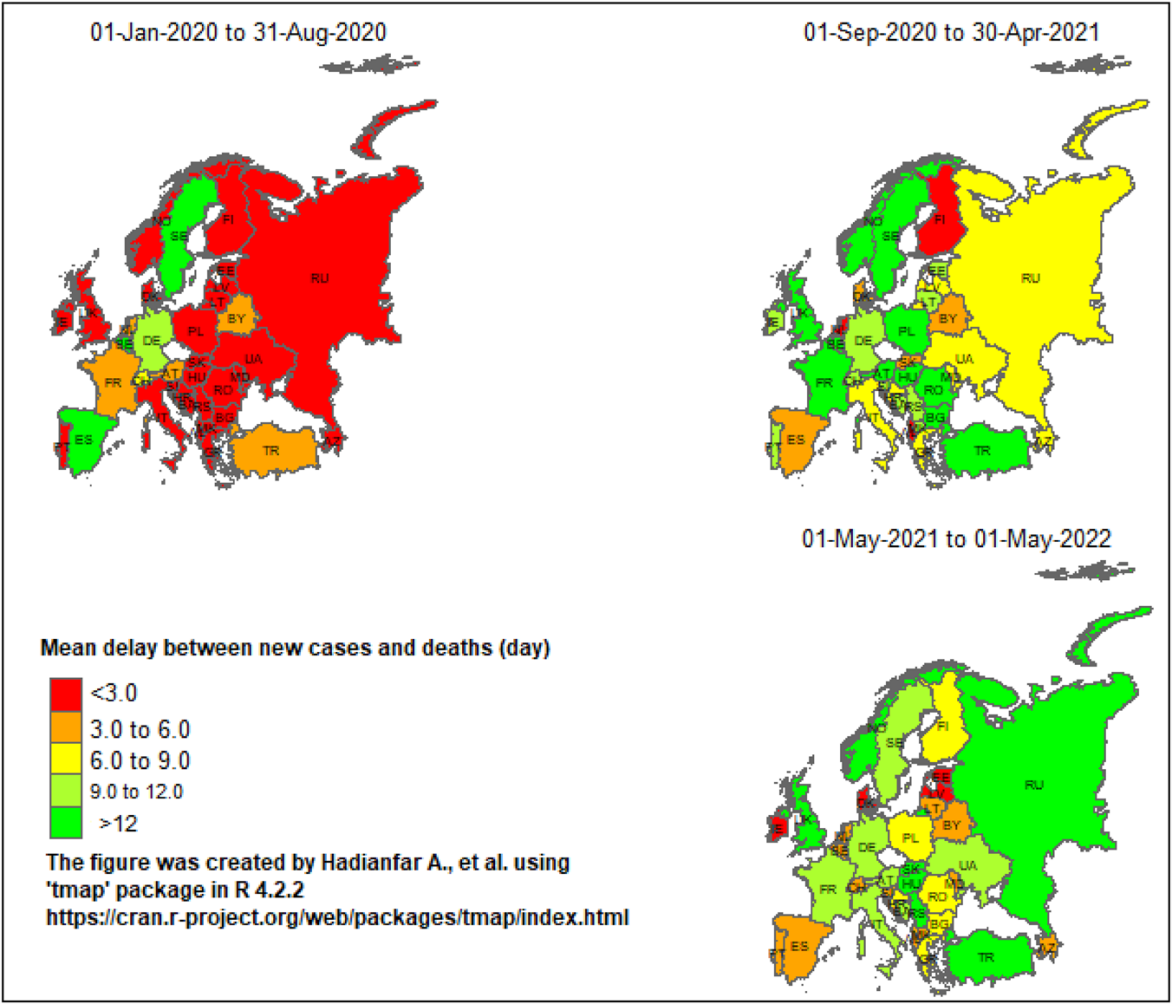
Spatial distribution of the mean delay between COVID-19 new cases and deaths among European countries from January 2020 to May 2022.

Figure 1 shows the spatial distribution of the mean delay for European countries at the three time intervals. The mean delay in the first peak of 2020 was the lowest in Eastern and Southern European countries, which increased in the next time intervals. In countries such as United Kingdom, Poland and France, this change was considerable. Also, the mean delay has remained unchanged in some countries, such as Germany and Sweden.

**Figure 2:**
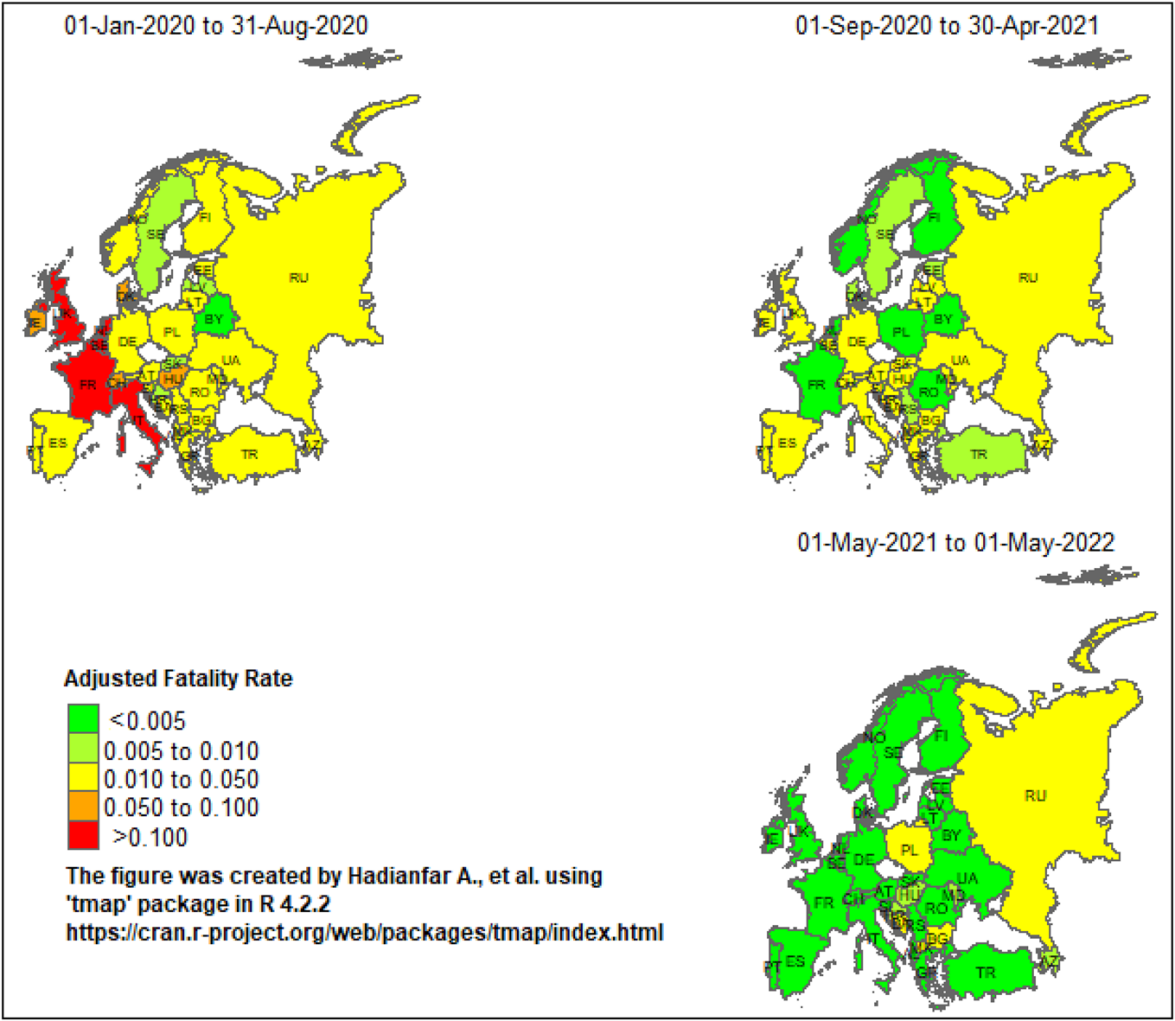
Spatial distribution of Adjusted Fatality Rate (AFR) of COVID-19 among European countries from January 2020 to May 2022.

Figure 2 shows the spatial distribution of the AFR concerning European countries in the first peak and subsequent peaks before and after vaccination. The AFRs in the first peak was the highest in some Western European countries, but it decreased in the subsequent peaks before vaccination. France, Italy, Belgium, Netherlands, and Britain experienced a significant decline in AFR during this period. However, countries like Germany and Ukraine maintained a consistent AFR. In 2021, most European countries had an AFR lower than 0.005. After vaccination the AFRs considerably decrese in most countries, but a few countries such as.

**Figure 3:**
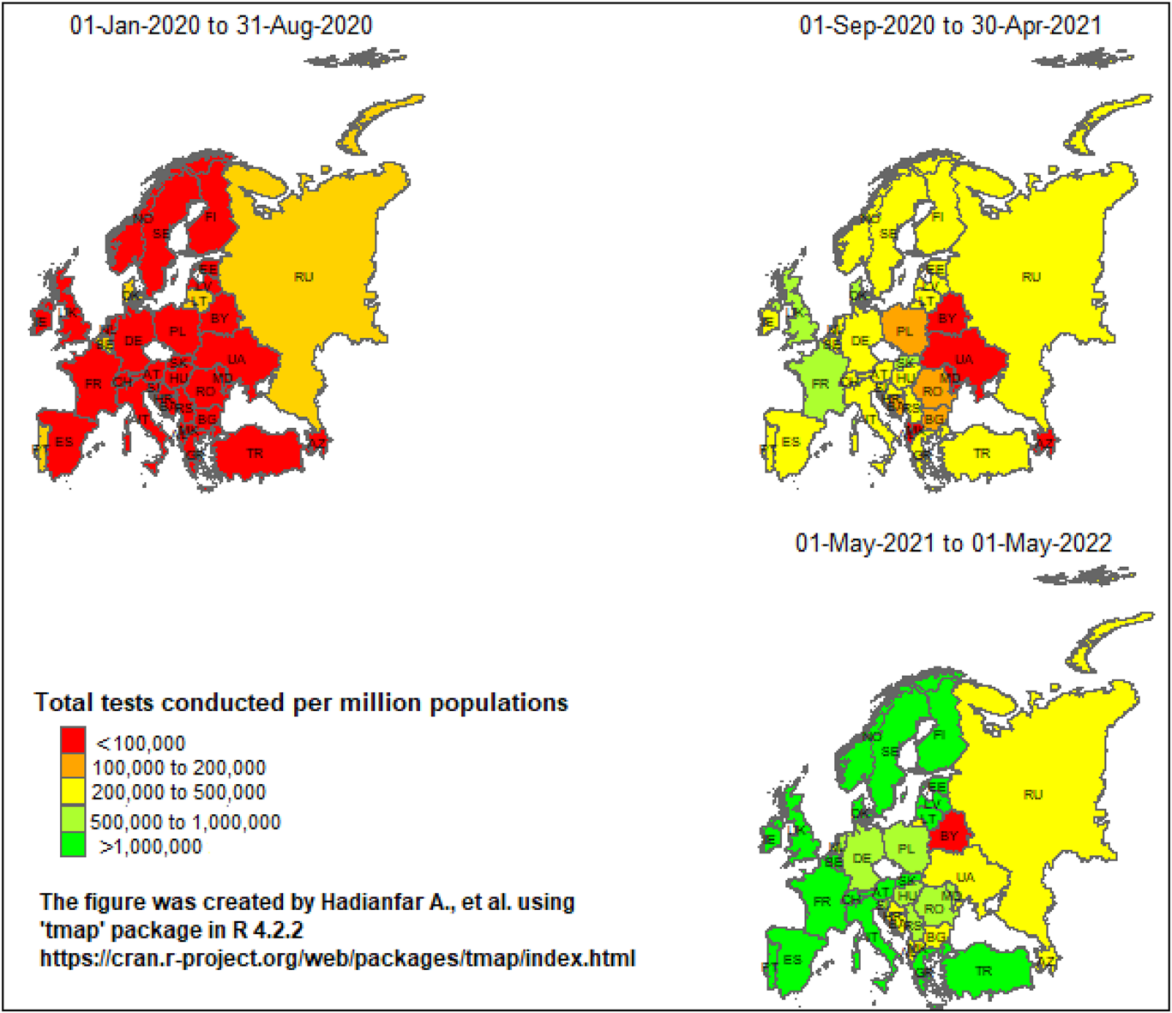
Spatial distribution of total COVID-19 tests conducted per million populations among European countries from January 2020 to May 2022.

Figure 3 displays the spatial distribution of the total number of COVID-19 tests conducted per million populations in European countries at the three time intervals. The total number of COVID-19 tests in European countries has generally increased from the first to the second time interval. In the first time interval, many European countries had conducted fewer than 100,000 tests per million populations. However, in the second interval, the total number of tests considerably increased, with the majority of European countries conducting 200,000 to 500,000 tests per million populations. This increasing trend continued in 2021 as well, so that most of the European countries had implemented more than one million tests per one million populations.

**Figure 4:**
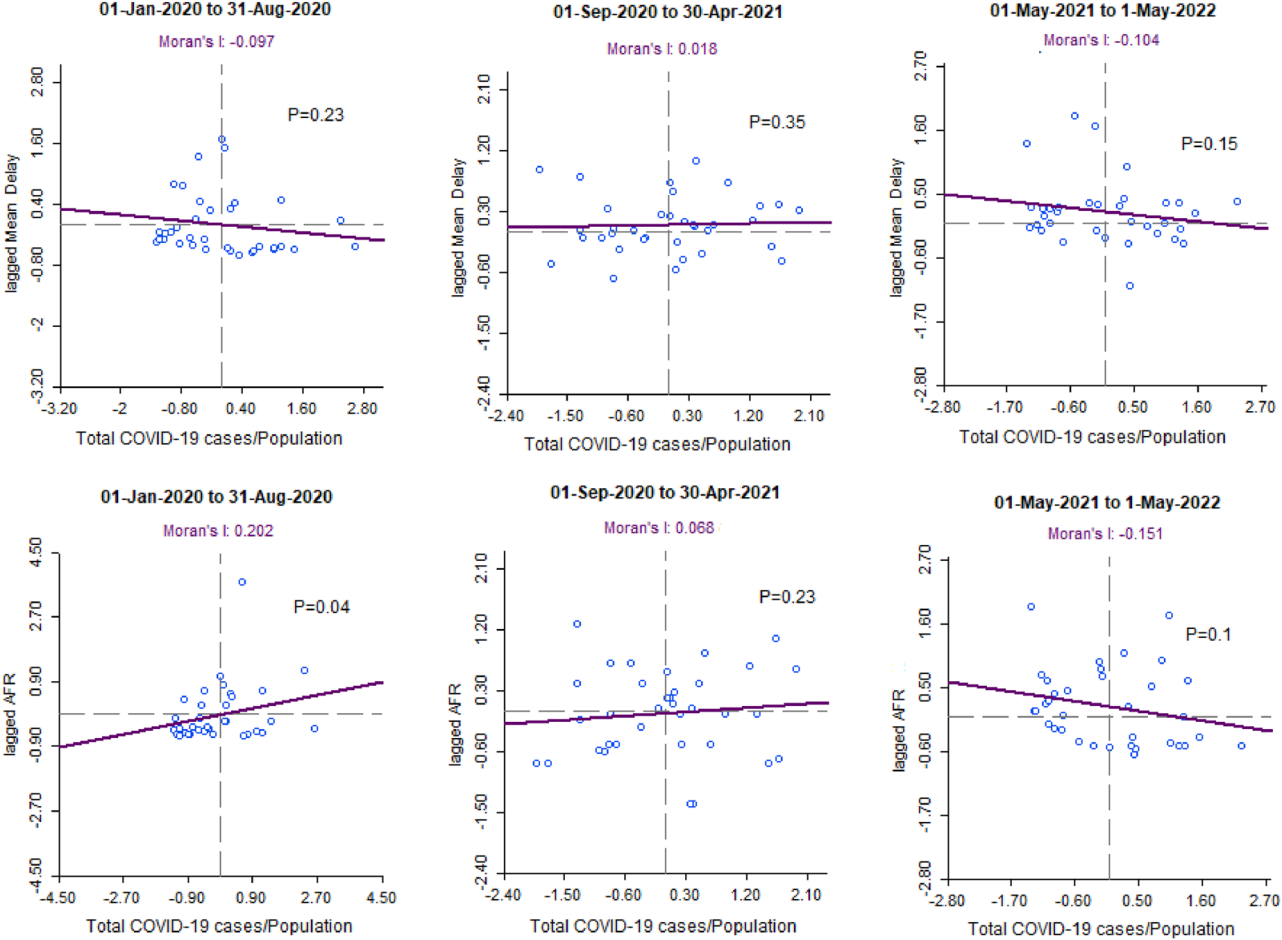
Plot of bivariate Moran’s I index for the spatial correlation of the mean delays and AFRs with rate of COVID-19 infections among neigboring countries from January 2020 to May 2022.

Figure 4 illustrates a significant positive spatial correlation between the AFRs and te rate of infection among neigboring countries (*p* = 0.04) at first peak of COVID-19.

## Discussion

Previous studies showed that there is a delay time between diagnosis, hospitalization, and death of COVID -19 by descriptive methods or ordinary regression^11–13,29,30^. Also, one study reported the lag time between the peak of incidence and fatality according to the density estimation^11^. In the present study, a novel application of DLMs was proposed and applied to estimate the mean delay between the change in the number of new cases and the number of death.

Ordinary estimation of fatality rate during a time interval for COVID -19 is biased due to delay effects of the new cases on death. For clarity the ordinary fatality rate since observing the first infection in UK on 24 Mar, 31 Mar, and 7 Apr (beginning the first outbreak) were 7.88%, 12.22, and 17.42%, respectively. And at the end of the first outbreak on 7Jul, 14Jul, and 21 Jul those were equal to 19.61%, 19.44%, and 19.23%, respectively^23^. So, the present study used the DLM to estimate the adjusted fatality rates according to delay effect of the new cases on death rate.

In our previous study DLM for Iranian COVID -19 hospitalized patients was used, and find that the mean delay between hospitalization and death was 5 days and the case fatality rate was 12 percent^8^. In present study, the mean delays and the adjusted fatality rates were heterogeneous among European countries. So, the models were fitted for each country, separately. The results of DLMs in present study show that in the second time interval, the mean delays considerably increased and the adjusted fatality rate decreased among most European countries. This result is desirable because after the first peak the number of COVID -19 tests for infection diagnosis and screening as well as medical resources, and proper management of medical resources increased in these countries. It is obvious that a part of this decline is due to diagnosis in the early phases of COVID -19 infection and an increasing in the delay between diagnoses to hospitalization. As a result, the health system has enough time to the preparation of treatment facilities for the future hospitalizations^9,15^. These results were supported with previous studies that reports the mean delays and fatality rates are different among countries and improved with superior health system conditions^13–16^. Also, the AFRs after vaccination were reduced considerably in most countries and a few countries with low percent of vaccination have increases in AFRs.

Spatial correlations of the mean delays were not significant at three time intervals. Only at the first peak of outbreak AFRs were significantly correlated with the rates of infection in neighboring countries. This result shows that except for the first peak, the transboundary infection was controlled among European countries.

In another study, Log-Linear DLM was used, and showed that the risk of death increased with the number of hospitalizations in past days and find the tolerance of hospitalization in Iran^9^. Also, a study reported that the fatality of COVID -19 increased with hospitalizations^15^. So, the fatality rate of COVID -19 was not constant and it is related to the health system tolerance.

DLMs are flexible and strongly appropriate to use in infectious diseases control decision making due to estimating important indices of different aspects of an outbreak.

Our proposed model is also appropriate for Socio-Epidemiologic studies since most of the epidemic outcomes are due to the previous change in the level of exposure or disease occurrence^31^, and consecutive outcomes will occur with delay. This delay plays a key role in estimating the gold time for decision-making regarding the allocation of resources and treatment facilities necessary to reduce fatality. Also, the epidemiologic indices such as fatality rates for these outcomes should be adjusted by the distributed lag models.

### Supplementary Information

Supplementary Information 1.

Supplementary Information 2.

Supplementary Information 3.

Supplementary Information 4.

## Data Availability

All data were used in this study are from two open access and open sources; the “ECDC” and “Our World in Data”. The copyright policy of ECDC is compatible with CC BY 4.0 license. And “Our World In Data” is a project of the Global Change Data Lab, a registered charity in England and Wales (Charity Number 1186433). The data and R-codes were used to this study are available respectively at supplementary files S3 & S4.
