# Supplementary material for: Application of distributed lag models and spatial analysis for comparing the performance of the COVID-19 control decisions in European countries
Source: Sci Rep. 2023 Oct 14;13:17466. doi: 10.1038/s41598-023-44830-z (PMC10576777; doi:10.1038/s41598-023-44830-z)
Supplement: Supplementary file 1 — Supplementary Information 1. [file 41598_2023_44830_MOESM1_ESM.docx]

**S1: Comparing Goodness of Fit Indices for Finite, Polynomial, and Koyck DLMs among European Countries COVID-19 Data, in 1 Jan 2020 to 31 Aug 2020 (a), 1 Sep 2020 to 30 Apr 2021 (b) 01 May 2021 to 1 May 2022 (c)**

| **S1: (a)** | **European Countries COVID-19 Data, 1 Jan 2020 to 31 Aug 2020** | | | | | | | | |
| --- | --- | --- | --- | --- | --- | --- | --- | --- | --- |
| **Country** | **Finite DLM** | | | **Polynomial DLM** | | | **Koyck DLM** | | |
|  | **AIC** | **MdAPE** | **Adjusted R^2^** | **AIC** | **MdAPE** | **Adjusted R^2^** | **AIC** | **MdAPE** | **Adjusted R^2^** |
| Albania | 607.6 | 0.27 | 0.72 | 595.5 | 0.26 | 0.72 | 606.2 | 0.14 | 0.74 |
| Austria | 940.8 | 0.21 | 0.7 | 944.7 | 0.18 | 0.67 | 1066.02 | 0.09 | 0.54 |
| Azerbaijan | 371.0 | 0.49 | 0.86 | 372.9 | 0.50 | 0.85 | 416.49 | 0.09 | 0.83 |
| Belarus | 530.5 | 3.93 | 0.84 | 644.4 | 4.02 | 0.66 | 754.57 | 0.20 | 0.51 |
| Belgium | 1363.7 | 0.50 | 0.97 | 1374.6 | 0.51 | 0.97 | 1377.92 | 0.36 | 0.98 |
| Bosnia and Herzegovina | 963.0 | 0.78 | 0.67 | 972.8 | 0.76 | 0.64 | 1049.4 | 0.34 | 0.59 |
| Bulgaria | 595.4 | 0.39 | 0.5 | 642.2 | 0.39 | 0.30 | 676.51 | 0.33 | 0.33 |
| Croatia | 475.4 | 0.05 | 0.43 | 487.1 | 0.05 | 0.34 | 533.32 | 0.04 | 0.26 |
| Cyprus | 137.4 | 0.40 | 0.26 | 164.5 | 0.43 | 0.06 | 161.59 | 0.18 | 0.07 |
| Czech IA | 642.5 | 1.08 | 0.75 | 656.2 | 1.16 | 0.71 | 770.78 | 0.37 | 0.57 |
| Denmark | 738.5 | 0.10 | 0.83 | 747.1 | 0.13 | 0.80 | 863.60 | 0.12 | 0.72 |
| Estonia | 355.6 | 0.57 | 0.5 | 385.6 | 0.52 | 0.36 | 455.90 | 0.19 | 0.14 |
| Finland | 909.7 | 3.20 | 0.65 | 927.6 | 2.80 | 0.60 | 931.9 | 0.45 | 0.60 |
| France | 2090.7 | 1.69 | 0.84 | 2137.2 | 2.00 | 0.77 | 2343.30 | 0.36 | 0.70 |
| Germany | 1521.7 | 0.62 | 0.92 | 1564.8 | 0.62 | 0.88 | 1712.06 | 0.36 | 0.86 |
| Greece | 542.5 | 0.46 | 0.56 | 549.5 | 0.46 | 0.51 | 613.97 | 0.17 | 0.42 |
| Hungary | 711.2 | 0.65 | 0.83 | 744.2 | 0.63 | 0.77 | 872.80 | 0.49 | 0.65 |
| Ireland | 1311.8 | 0.38 | 0.71 | 1397.0 | 0.38 | 0.48 | 1559.55 | 0.22 | 0.26 |
| Italy | 1755.5 | 0.11 | 0.97 | 1758.3 | 0.10 | 0.96 | 1957.33 | 0.11 | 0.95 |
| Kosovo | 942.2 | 0.67 | 0.77 | 959.0 | 0.64 | 0.74 | 1059.9 | 0.40 | 0.67 |
| Latvia | 236.8 | 2.47 | 0.22 | 253.9 | 2.38 | 0.08 | 272.64 | 0.37 | 0.01 |
| Lithuania | 326.2 | 0.46 | 0.21 | 317.0 | 0.43 | 0.20 | 364.07 | 0.26 | 0.03 |
| Moldova | 768.4 | 0.60 | 0.67 | 787.2 | 0.54 | 0.61 | 891.16 | 0.17 | 0.48 |
| Netherlands | 1437.2 | 0.07 | 0.89 | 1501.3 | 0.05 | 0.83 | 1638.61 | 0.06 | 0.80 |
| North Macedonia | 587.5 | 0.24 | 0.73 | 607.5 | 0.23 | 0.68 | 670.58 | 0.16 | 0.63 |
| Norway | 707.3 | 0.99 | 0.55 | 709.8 | 2.25 | 0.51 | 795.94 | 1.08 | 0.38 |
| Poland | 1091.0 | 1.06 | 0.59 | 1070.7 | 1.31 | 0.61 | 1106.69 | 0.04 | 0.69 |
| Portugal | 1055.1 | 1.72 | 0.77 | 1055.2 | 1.71 | 0.75 | 1127.76 | 0.23 | 0.75 |
| Romania | 1011.6 | 0.69 | 0.78 | 1012.5 | 0.69 | 0.77 | 1137.33 | 0.01 | 0.69 |
| Russia | 1535.2 | 0.54 | 0.88 | 1527.0 | 0.59 | 0.87 | 1588.50 | 0.21 | 0.90 |
| Serbia | 704.5 | 0.27 | 0.56 | 729.7 | 0.26 | 0.45 | 749.59 | 0.14 | 0.53 |
| Slovakia | 183.6 | 0.49 | 0.44 | 204.6 | 0.47 | 0.32 | 280.15 | 0.13 | 0.01 |
| Slovenia | 388.5 | 0.21 | 0.71 | 424.9 | 0.18 | 0.60 | 516.27 | 0.09 | 0.42 |
| Spain | 1514.3 | 0.49 | 0.99 | 1533.4 | 0.50 | 0.99 | 1581.80 | 0.09 | 0.99 |
| Sweden | 2095.3 | 3.93 | 0.27 | 2077.4 | 4.02 | 0.29 | 1542.5 | 0.20 | 0.96 |
| Switzerland | 872.3 | 0.50 | 0.97 | 904.5 | 0.51 | 0.95 | 1025.69 | 0.36 | 0.94 |
| Turkey | 1163.2 | 0.78 | 0.96 | 1148.2 | 0.76 | 0.95 | 988.00 | 0.34 | 0.99 |
| Ukraine | 879.6 | 0.39 | 0.81 | 874.9 | 0.39 | 0.80 | 926.67 | 0.33 | 0.81 |
| United Kingdom | 2037.5 | 0.05 | 0.89 | 2111.3 | 0.05 | 0.82 | 2218.23 | 0.04 | 0.86 |

| **S1: (b)** | **European Countries COVID-19 Data, 1 Sep 2020 to 30 Apr 2021** | | | | | | | | |
| --- | --- | --- | --- | --- | --- | --- | --- | --- | --- |
| **Countries** | **Finite DLM Model** | | | **polynomial DLM model** | | | **koyck DLM model** | | |
|  | **AIC** | **MdAPE** | **Adjusted R^2^** | **AIC** | **MdAPE** | **Adjusted R^2^** | **AIC** | **MdAPE** | **Adjusted R^2^** |
| Albania | 1372.4 | 0.30 | 0.85 | 1469.99 | 0.30 | 0.72 | 1594.77 | 0.08 | 0.69 |
| Austria | 1421.3 | 0.64 | 0.86 | 1419.70 | 0.65 | 0.85 | 1250.80 | 0.05 | 0.97 |
| Azerbaijan | 1382.7 | 0.11 | 0.55 | 1381.60 | 0.12 | 0.52 | 1428.52 | 0.03 | 0.63 |
| Belarus | 979.3 | 0.60 | 0.96 | 970.80 | 0.59 | 0.95 | 894.00 | 0.02 | 0.98 |
| Belgium | 1285.9 | 0.34 | 0.92 | 1302.50 | 0.33 | 0.91 | 1328.90 | 0.12 | 0.93 |
| Bosnia and Herzegovina | 1270.3 | 0.46 | 0.80 | 1297.50 | 0.48 | 0.75 | 1478.30 | 0.24 | 0.60 |
| Bulgaria | 790.9 | 0.45 | 0.33 | 805.93 | 0.48 | 0.22 | 903.02 | 0.07 | 0.00 |
| Croatia | 12.3 | 0.46 | 0.44 | 42.57 | 0.47 | 0.30 | 28.64 | 0.37 | 0.30 |
| Cyprus | 618.5 | 0.39 | 0.57 | 634.60 | 0.39 | 0.49 | 661.60 | 0.02 | 0.52 |
| Czech IA | 1510.9 | 0.67 | 0.90 | 1493.70 | 0.70 | 0.90 | 1425.41 | 0.13 | 0.96 |
| Denmark | 1952.1 | 0.54 | 0.88 | 2026.40 | 0.53 | 0.79 | 2177.84 | 0.28 | 0.79 |
| Estonia | 2042.0 | 0.45 | 0.84 | 2084.60 | 0.45 | 0.78 | 2235.30 | 0.29 | 0.78 |
| Finland | 281.8 | 0.45 | 0.22 | 246.50 | 0.50 | 0.09 | 266.00 | 0.37 | 0.06 |
| France | 933.7 | 0.54 | 0.95 | 996.02 | 0.52 | 0.92 | 1158.20 | 0.20 | 0.86 |
| Germany | -282.6 | 0.45 | 0.14 | -275.94 | 0.47 | 0.05 | -301.20 | 0.04 | -0.01 |
| Greece | 953.9 | 0.46 | 0.67 | 939.20 | 0.46 | 0.68 | 1056.60 | 0.02 | 0.58 |
| Hungary | 2220.7 | 0.63 | 0.65 | 2312.10 | 0.65 | 0.36 | 2508.80 | 0.06 | 0.25 |
| Ireland | 42.9 | 0.42 | 0.40 | 75.80 | 0.41 | 0.22 | 105.90 | 0.20 | 0.04 |
| Italy | 1724.0 | 0.43 | 0.87 | 1716.20 | 0.47 | 0.86 | 1631.06 | 0.31 | 0.96 |
| Kosovo | 1168.9 | 0.27 | 0.59 | 1153.5 | 0.28 | 0.59 | 1246.4 | 0.03 | 0.53 |
| Latvia | 249.5 | 0.53 | 0.41 | 263.50 | 0.59 | 0.32 | 282.70 | 0.18 | 0.27 |
| Lithuania | 1421.9 | 0.66 | 0.93 | 1477.90 | 0.83 | 0.89 | 1791.50 | 1.05 | 0.65 |
| Moldova | 760.2 | 0.24 | 0.55 | 814.02 | 0.24 | 0.34 | 843.90 | 0.08 | 0.41 |
| Netherlands | 379.9 | 0.55 | 0.60 | 389.73 | 0.53 | 0.54 | 407.07 | 0.12 | 0.55 |
| North Macedonia | 960.9 | 0.45 | 0.87 | 971.30 | 0.44 | 0.85 | 1166.60 | 0.06 | 0.70 |
| Norway | 1187.2 | 0.51 | 0.87 | 1182.20 | 0.49 | 0.86 | 1197.98 | 0.09 | 0.90 |
| Poland | 1544.5 | 0.52 | 0.91 | 1547.80 | 0.56 | 0.90 | 1689.90 | 0.05 | 0.89 |
| Portugal | 1299.1 | 0.46 | 0.90 | 1281.60 | 0.47 | 0.90 | 1256.40 | 0.06 | 0.95 |
| Romania | 1927.7 | 0.42 | 0.95 | 1959.24 | 0.43 | 0.93 | 2125.30 | 0.02 | 0.92 |
| Russia | 870.1 | 0.35 | 0.79 | 910.50 | 0.30 | 0.71 | 1031.98 | 0.09 | 0.58 |
| Serbia | 1975.0 | 0.30 | 0.88 | 2017.00 | 0.30 | 0.83 | 2190.20 | 0.08 | 0.80 |
| Slovakia | 281.5 | 0.57 | 0.37 | 301.20 | 0.56 | 0.20 | 294.90 | 0.04 | 0.30 |
| Slovenia | 1693.6 | 0.64 | 0.96 | 1760.26 | 0.65 | 0.94 | 1910.96 | 0.05 | 0.94 |
| Spain | 701.1 | 0.11 | 0.90 | 724.77 | 0.12 | 0.88 | 832.70 | 0.03 | 0.83 |
| Sweden | -403.7 | 0.60 | 0.16 | -387.20 | 0.59 | 0.01 | -425.80 | 0.02 | 0.10 |
| Switzerland | 1502.3 | 0.34 | 0.94 | 1613.50 | 0.33 | 0.88 | 1794.80 | 0.12 | 0.83 |
| Turkey | 1665.2 | 0.46 | 0.78 | 1657.80 | 0.48 | 0.77 | 1377.70 | 0.24 | 0.98 |
| Ukraine | 880.1 | 0.45 | 0.29 | 884.80 | 0.48 | 0.22 | 941.80 | 0.07 | 0.22 |
| United Kingdom | 948.2 | 0.46 | 0.45 | 930.50 | 0.47 | 0.47 | 994.85 | 0.37 | 0.47 |

| **S1: (c)** | **European Countries COVID-19 Data May 2021 to 1 May 2022** | | | | | | | | |
| --- | --- | --- | --- | --- | --- | --- | --- | --- | --- |
| **Countries** | **Finite DLM Model** | | | **polynomial DLM model** | | | **koyck DLM model** | | |
|  | **AIC** | **MSE** | **Adjusted R^2^** | **AIC** | **MSE** | **Adjusted R^2^** | **AIC** | **MSE** | **Adjusted R^2^** |
| Albania | 1673.3 | 0.52 | 0.36 | 1667.5 | 0.51 | 0.35 | 1474.3 | 0.11 | 0.66 |
| Austria | 2948.3 | 0.56 | 0.24 | 2929.7 | 0.55 | 0.26 | 2380.3 | 0.05 | 0.87 |
| Azerbaijan | 2314.9 | 0.47 | 0.67 | 2299.9 | 0.48 | 0.68 | 2083.0 | 0.07 | 0.86 |
| Belarus | 1872.2 | 0.16 | 0.20 | 1859.8 | 0.16 | 0.20 | 1561.4 | 0.03 | 0.70 |
| Belgium | 2607.3 | 0.45 | 0.53 | 2607.4 | 0.46 | 0.52 | 2408.4 | 0.08 | 0.79 |
| Bosnia and Herzegovina | 2690.5 | 0.47 | 0.61 | 2753.9 | 0.49 | 0.52 | 2906.0 | 0.21 | 0.45 |
| Bulgaria | 3592.5 | 0.55 | 0.51 | 3662.0 | 0.56 | 0.38 | 3832.0 | 0.30 | 0.31 |
| Croatia | 2702.3 | 0.42 | 0.73 | 2687.6 | 0.43 | 0.71 | 2440.0 | 0.06 | 0.89 |
| Cyprus | 1397.5 | 0.48 | 0.26 | 1419.9 | 0.45 | 0.20 | 1460.6 | 0.31 | 0.20 |
| Czech IA | 3343.1 | 0.67 | 0.35 | 3324.8 | 0.69 | 0.36 | 2556.0 | 0.03 | 0.94 |
| Denmark | 2540.5 | 0.80 | 0.57 | 2539.9 | 0.80 | 0.56 | 2552.6 | 0.27 | 0.64 |
| Estonia | 1773.7 | 0.56 | 0.23 | 1761.5 | 0.53 | 0.24 | 1798.1 | 0.31 | 0.32 |
| Finland | 2259.6 | 0.44 | 0.76 | 2276.5 | 0.45 | 0.77 | 2358.6 | 0.21 | 0.74 |
| France | 3842.6 | 0.37 | 0.69 | 4059.5 | 1.08 | 0.45 | 4166.6 | 0.22 | 0.46 |
| Germany | 4287.2 | 0.57 | 0.37 | 4395.3 | 0.96 | 0.18 | 3998.3 | 0.09 | 0.79 |
| Greece | 3225.4 | 0.32 | 0.39 | 3209.3 | 0.33 | 0.40 | 2886.1 | 0.05 | 0.82 |
| Hungary | 3457.6 | 0.65 | 0.60 | 3440.0 | 0.66 | 0.61 | 2943.0 | 0.04 | 0.93 |
| Ireland | 2025.8 | 0.44 | 0.25 | 2011.0 | 0.42 | 0.25 | 1930.3 | 0.16 | 0.51 |
| Italy | 3540.3 | 0.38 | 0.87 | 3763.8 | 0.59 | 0.76 | 3676.1 | 0.05 | 0.87 |
| Kosovo | 2087.9 | 0.70 | 0.38 | 2074.3 | 0.75 | 0.38 | 1738.8 | 0.12 | 0.79 |
| Latvia | 2675.5 | 0.68 | 0.11 | 2660.0 | 0.70 | 0.11 | 2546.0 | 0.23 | 0.49 |
| Lithuania | 2632.3 | 0.53 | 0.12 | 2612.5 | 0.53 | 0.11 | 2264.4 | 0.08 | 0.74 |
| Moldova | 2778.6 | 0.69 | 0.26 | 2760.9 | 0.70 | 0.28 | 2493.6 | 0.12 | 0.73 |
| Netherlands | 2962.6 | 0.84 | 0.03 | 2938.7 | 0.84 | 0.01 | 2644.9 | 0.15 | 0.62 |
| North Macedonia | 2357.9 | 0.50 | 0. 48 | 2339.4 | 0.50 | 0.49 | 2255.0 | 0.11 | 0.67 |
| Norway | 3060.0 | 4.97 | 0.49 | 3232.8 | 5.90 | 0.10 | 3372.3 | 7.63 | 0.01 |
| Poland | 4348.2 | 1.01 | 0.58 | 4403.1 | 1.03 | 0.50 | 4520.4 | 0.68 | 0.56 |
| Portugal | 2212.2 | 0.36 | 0.40 | 2845.5 | 0.36 | 0.39 | 2228.7 | 0.08 | 0.83 |
| Romania | 4288.4 | 0.71 | 0.24 | 4272.0 | 0.73 | 0.25 | 3932.9 | 0.09 | 0.80 |
| Russia | 4988.3 | 0.27 | 0.01 | 4995.4 | 0.77 | 0.72 | 3377.2 | 0.00 | 0.99 |
| Serbia | 2810.0 | 0.51 | 0.64 | 2803.3 | 0.53 | 0.63 | 1773.7 | 0.02 | 0.98 |
| Slovakia | 3133.7 | 0.69 | 0.25 | 3118.0 | 0.67 | 0.25 | 2559.0 | 0.04 | 0.88 |
| Slovenia | 2108.1 | 0.53 | 0.40 | 2092.3 | 0.52 | 0.41 | 1898.2 | 0.12 | 0.71 |
| Spain | 3581.2 | 0.43 | 0.69 | 3573.0 | 0.45 | 0.69 | 3519.8 | 0.08 | 0.81 |
| Sweden | 2564.7 | 0.71 | 0.63 | 2560.5 | 0.73 | 0.62 | 2197.6 | 0.07 | 0.89 |
| Switzerland | 2315.4 | 0.42 | 0.27 | 2299.5 | 0.43 | 0.28 | 2064.0 | 0.11 | 0.69 |
| Turkey | 3950.6 | 0.35 | 0.27 | 3927.8 | 0.35 | 0.30 | 3119.2 | 0.02 | 0.95 |
| Ukraine | 4547.1 | 0.61 | 0.43 | 4544.8 | 0.61 | 0.42 | 4265.7 | 0.07 | 0.82 |
| United Kingdom | 3610.5 | 0.28 | 0.56 | 3656.4 | 0.57 | 0.53 | 3035.9 | 0.02 | 0.94 |
