# Supplementary material for: Application of distributed lag models and spatial analysis for comparing the performance of the COVID-19 control decisions in European countries
Source: Sci Rep. 2023 Oct 14;13:17466. doi: 10.1038/s41598-023-44830-z (PMC10576777; doi:10.1038/s41598-023-44830-z)
Supplement: Supplementary file 2 — Supplementary Information 2. [file 41598_2023_44830_MOESM2_ESM.docx]

**S2: Forecasting new death of COVID-19 based on the Koyck DLM model from 1 to 14 Sep 2020 among European Countries**

| Country | New death | **9/1/20** | **9/2/20** | **9/3/20** | **9/4/20** | **9/5/20** | **9/6/20** | **9/7/20** | **9/8/20** | **9/9/20** | **9/10/20** | **9/11/20** | **9/12/20** | **9/13/20** | **9/14/20** |
| --- | --- | --- | --- | --- | --- | --- | --- | --- | --- | --- | --- | --- | --- | --- | --- |
| Albania | Observed | 5 | 4 | 6 | 6 | 5 | 5 | 6 | 4 | 3 | 2 | 1 | 2 | 3 | 3 |
|  | Forecast | 2 | 2 | 2 | 2 | 3 | 2 | 2 | 2 | 2 | 2 | 2 | 3 | 3 | 3 |
|  | Lower | 1 | 1 | 0 | 0 | 1 | 0 | 1 | 0 | 1 | 0 | 1 | 1 | 1 | 1 |
|  | Upper | 4 | 4 | 3 | 4 | 4 | 4 | 4 | 4 | 4 | 4 | 4 | 4 | 5 | 5 |
| Austria | real | 0 | 3 | 2 | 2 | 1 | 5 | 1 | 2 | 0 | 1 | 1 | 4 | 4 | 4 |
|  | forecast | 0 | 1 | 1 | 1 | 2 | 2 | 2 | 2 | 2 | 3 | 3 | 3 | 3 | 3 |
|  | Lower | -5 | -6 | -6 | -7 | -6 | -7 | -6 | -6 | -6 | -5 | -6 | -6 | -6 | -6 |
|  | Upper | 5 | 7 | 8 | 9 | 9 | 9 | 10 | 10 | 11 | 11 | 11 | 12 | 11 | 11 |
| Azerbaijan | real | 2 | 3 | 2 | 2 | 3 | 2 | 2 | 3 | 3 | 1 | 3 | 1 | 1 | 2 |
|  | forecast | 6 | 7 | 8 | 8 | 8 | 8 | 8 | 8 | 8 | 8 | 8 | 8 | 8 | 8 |
|  | Lower | 3 | 5 | 6 | 6 | 6 | 5 | 5 | 5 | 5 | 5 | 6 | 5 | 6 | 5 |
|  | Upper | 8 | 10 | 10 | 11 | 11 | 11 | 10 | 10 | 10 | 10 | 10 | 10 | 10 | 10 |
| Belarus | real | 5 | 5 | 5 | 5 | 5 | 4 | 6 | 5 | 5 | 5 | 6 | 6 | 6 | 6 |
|  | forecast | 5 | 4 | 4 | 4 | 4 | 3 | 3 | 3 | 3 | 3 | 3 | 3 | 3 | 3 |
|  | Lower | 3 | 2 | 1 | 0 | 0 | 0 | 0 | 0 | 0 | -1 | -1 | -1 | -1 | -1 |
|  | Upper | 7 | 7 | 7 | 7 | 7 | 7 | 7 | 7 | 7 | 7 | 7 | 7 | 7 | 6 |
| Belgium | real | 1 | 3 | 3 | 3 | 4 | 2 | 1 | 3 | 4 | 3 | 2 | 3 | 5 | 1 |
|  | forecast | 5 | 5 | 4 | 3 | 3 | 1 | 0 | 0 | 0 | 0 | 0 | 0 | 0 | 0 |
|  | Lower | -15 | -25 | -30 | -34 | -34 | -37 | -41 | -42 | -44 | -45 | -47 | -46 | -51 | -53 |
|  | Upper | 26 | 33 | 35 | 39 | 39 | 41 | 42 | 40 | 43 | 44 | 46 | 49 | 46 | 44 |
| Bosnia and Herzegovina | real | 11 | 11 | 7 | 9 | 3 | 12 | 4 | 9 | 5 | 6 | 5 | 6 | 4 | 6 |
|  | forecast | 4 | 4 | 4 | 5 | 6 | 5 | 3 | 4 | 5 | 6 | 7 | 8 | 6 | 4 |
|  | Lower | -1 | -1 | -1 | 0 | 2 | 0 | -2 | -1 | 0 | 0 | 2 | 2 | 1 | -2 |
|  | Upper | 8 | 9 | 9 | 9 | 11 | 10 | 8 | 8 | 10 | 10 | 12 | 12 | 11 | 9 |
| Bulgaria | real | 6 | 10 | 7 | 6 | 5 | 1 | 15 | 10 | 4 | 7 | 4 | 3 | 9 | 7 |
|  | forecast | 8 | 6 | 6 | 4 | 5 | 5 | 6 | 7 | 7 | 6 | 4 | 5 | 6 | 6 |
|  | Lower | 3 | 0 | -1 | -1 | -1 | 0 | 0 | 2 | 2 | 0 | -1 | -1 | 0 | 1 |
|  | Upper | 14 | 12 | 12 | 9 | 10 | 11 | 11 | 12 | 14 | 12 | 10 | 10 | 11 | 12 |
| Croatia | real | 2 | 1 | 4 | 3 | 1 | 2 | 1 | 3 | 2 | 3 | 2 | 3 | 7 | 6 |
|  | forecast | 1 | 1 | 1 | 1 | 1 | 1 | 1 | 1 | 1 | 1 | 1 | 1 | 1 | 1 |
|  | Lower | -1 | -2 | -1 | -1 | -1 | -1 | -2 | -2 | -2 | -1 | -1 | -1 | -2 | -1 |
|  | Upper | 3 | 3 | 4 | 4 | 3 | 3 | 3 | 3 | 3 | 4 | 4 | 4 | 3 | 3 |
| Cyprus | real | 0 | 0 | 0 | 0 | 0 | 0 | 0 | 0 | 0 | 0 | 0 | 0 | 0 | 0 |
|  | forecast | 0 | 0 | 0 | 0 | 0 | 0 | 0 | 0 | 0 | 0 | 0 | 0 | 0 | 0 |
|  | Lower | -1 | -1 | -1 | -1 | -1 | -1 | -1 | -1 | -1 | -1 | -1 | -1 | -1 | -1 |
|  | Upper | 1 | 1 | 1 | 1 | 1 | 1 | 1 | 1 | 1 | 1 | 1 | 1 | 1 | 1 |
| Czechia | real | 1 | 1 | 0 | 4 | 3 | 5 | 3 | 2 | 5 | 3 | 5 | 3 | 6 | 5 |
|  | forecast | 1 | 1 | 2 | 2 | 2 | 2 | 2 | 2 | 2 | 2 | 2 | 2 | 2 | 2 |
|  | Lower | -2 | -3 | -3 | -2 | -3 | -3 | -3 | -3 | -3 | -3 | -3 | -3 | -3 | -3 |
|  | Upper | 4 | 5 | 6 | 6 | 7 | 7 | 6 | 6 | 7 | 6 | 7 | 7 | 7 | 7 |
| Denmark | real | 0 | 1 | 1 | 0 | 1 | 0 | 0 | 1 | 0 | 0 | 1 | 0 | 1 | 1 |
|  | forecast | 0 | 0 | 0 | 0 | 0 | 0 | 0 | 0 | 0 | 0 | 0 | 0 | 0 | 0 |
|  | Lower | -4 | -5 | -5 | -5 | -5 | -5 | -6 | -6 | -7 | -6 | -5 | -5 | -5 | -4 |
|  | Upper | 4 | 5 | 5 | 6 | 5 | 5 | 5 | 5 | 5 | 4 | 5 | 6 | 6 | 6 |
| Estonia | real | 0 | 0 | 0 | 0 | 0 | 0 | 0 | 0 | 0 | 0 | 0 | 0 | 0 | 0 |
|  | forecast | 0 | 0 | 0 | 0 | 0 | 0 | 0 | 0 | 0 | 0 | 0 | 0 | 0 | 0 |
|  | Lower | -2 | -1 | -1 | -1 | -1 | -1 | -1 | -1 | -1 | -1 | -1 | -1 | -2 | -2 |
|  | Upper | 2 | 2 | 2 | 1 | 2 | 2 | 2 | 2 | 2 | 2 | 2 | 2 | 1 | 2 |
| Finland | real | 0 | 1 | 1 | 0 | 0 | 1 | 0 | 1 | 0 | 0 | 3 | 0 | 2 | 0 |
|  | forecast | 0 | 0 | 0 | 0 | 0 | 0 | 0 | 0 | 0 | 0 | 0 | 0 | 0 | 0 |
|  | Lower | -3 | -3 | -4 | -4 | -4 | -4 | -4 | -4 | -4 | -4 | -4 | -4 | -4 | -4 |
|  | Upper | 4 | 4 | 4 | 4 | 4 | 3 | 4 | 4 | 4 | 3 | 4 | 4 | 3 | 3 |
| France | real | 26 | 25 | 20 | 12 | 4 | 23 | 38 | 32 | 19 | 80 | 18 | 5 | 34 | 50 |
|  | forecast | 33 | 38 | 38 | 28 | 47 | 46 | 55 | 53 | 58 | 50 | 38 | 64 | 50 | 56 |
|  | Lower | -257 | -331 | -344 | -411 | -394 | -399 | -415 | -393 | -377 | -393 | -417 | -418 | -408 | -453 |
|  | Upper | 315 | 359 | 386 | 406 | 468 | 491 | 502 | 523 | 508 | 493 | 481 | 578 | 495 | 508 |
| Germany | real | 12 | 9 | 9 | 7 | 2 | 4 | 8 | 12 | 12 | 6 | 6 | 3 | 1 | 4 |
|  | forecast | 5 | 7 | 9 | 11 | 12 | 13 | 14 | 15 | 16 | 17 | 17 | 18 | 18 | 18 |
|  | Lower | -44 | -59 | -76 | -89 | -84 | -91 | -98 | -96 | -90 | -86 | -87 | -81 | -83 | -83 |
|  | Upper | 53 | 75 | 82 | 92 | 98 | 106 | 115 | 113 | 126 | 123 | 121 | 121 | 121 | 121 |
| Greece | real | 5 | 2 | 5 | 1 | 1 | 4 | 5 | 1 | 3 | 4 | 3 | 2 | 3 | 5 |
|  | forecast | 2 | 2 | 1 | 1 | 1 | 1 | 1 | 1 | 1 | 1 | 1 | 1 | 1 | 1 |
|  | Lower | 0 | -1 | -2 | -2 | -2 | -2 | -2 | -2 | -2 | -2 | -2 | -2 | -2 | -2 |
|  | Upper | 5 | 5 | 4 | 4 | 4 | 4 | 4 | 4 | 4 | 4 | 4 | 4 | 4 | 4 |
| Hungary | real | 1 | 1 | 3 | 1 | 1 | 3 | 0 | 1 | 1 | 2 | 2 | 1 | 2 | 4 |
|  | forecast | 0 | 1 | 1 | 1 | 1 | 1 | 1 | 1 | 1 | 1 | 1 | 1 | 1 | 1 |
|  | Lower | -5 | -5 | -5 | -6 | -6 | -6 | -6 | -6 | -6 | -6 | -5 | -6 | -6 | -5 |
|  | Upper | 6 | 7 | 7 | 7 | 8 | 8 | 8 | 8 | 8 | 8 | 7 | 7 | 8 | 7 |
| Ireland | real | 0 | 0 | 0 | 0 | 1 | 0 | 0 | 2 | 1 | 1 | 1 | 0 | 3 | 2 |
|  | forecast | 1 | 0 | 0 | 0 | 0 | 0 | 0 | 0 | 0 | 0 | 0 | 1 | 1 | 1 |
|  | Lower | -9 | -10 | -10 | -10 | -10 | -10 | -9 | -11 | -11 | -10 | -10 | -9 | -10 | -10 |
|  | Upper | 11 | 11 | 11 | 10 | 11 | 11 | 11 | 10 | 11 | 11 | 11 | 11 | 11 | 12 |
| Italy | real | 6 | 8 | 6 | 10 | 11 | 15 | 8 | 12 | 10 | 14 | 10 | 10 | 6 | 7 |
|  | forecast | 5 | 8 | 10 | 13 | 16 | 15 | 15 | 12 | 13 | 14 | 18 | 17 | 18 | 15 |
|  | Lower | 11 | 11 | 11 | 10 | 11 | 11 | 11 | 10 | 11 | 11 | 11 | 11 | 11 | 12 |
|  | Upper | -91 | -125 | -122 | -119 | -127 | -120 | -118 | -118 | -115 | -105 | -122 | -128 | -107 | -122 |
| Kosovo | real | 9 | 8 | 6 | 9 | 3 | 5 | 7 | 5 | 0 | 6 | 7 | 5 | 3 | 7 |
|  | forecast | 4 | 5 | 3 | 4 | 7 | 6 | 8 | 8 | 9 | 9 | 9 | 9 | 9 | 8 |
|  | Lower | 0 | 0 | -2 | 0 | 2 | 2 | 3 | 3 | 4 | 4 | 4 | 3 | 4 | 4 |
|  | Upper | 9 | 9 | 8 | 9 | 12 | 11 | 12 | 13 | 13 | 13 | 13 | 13 | 14 | 13 |
| Latvia | real | 0 | 0 | 0 | 1 | 0 | 0 | 0 | 0 | 0 | 0 | 0 | 0 | 0 | 0 |
|  | forecast | 0 | 0 | 0 | 0 | 0 | 0 | 0 | 0 | 0 | 0 | 0 | 0 | 0 | 0 |
|  | Lower | -1 | -1 | -1 | -1 | -1 | -1 | -1 | -1 | -1 | -1 | -1 | -1 | -1 | -1 |
|  | Upper | 1 | 1 | 1 | 1 | 1 | 1 | 1 | 1 | 1 | 1 | 1 | 1 | 1 | 1 |
| Lithuania | real | 0 | 0 | 0 | 0 | 0 | 0 | 0 | 0 | 0 | 0 | 1 | 0 | 1 | 0 |
|  | forecast | 0 | 0 | 0 | 0 | 0 | 0 | 0 | 0 | 0 | 0 | 0 | 0 | 0 | 0 |
|  | Lower | -1 | -1 | -1 | -1 | -1 | -1 | -1 | -1 | -1 | -1 | -1 | -1 | -1 | -1 |
|  | Upper | 1 | 1 | 1 | 1 | 1 | 1 | 1 | 1 | 1 | 2 | 1 | 1 | 1 | 1 |
| Netherlands | real | 8 | 5 | 0 | 2 | 4 | 2 | 0 | 1 | 2 | 3 | 3 | 1 | 1 | 2 |
|  | forecast | 1 | 1 | 1 | 1 | 0 | 0 | 0 | 0 | 0 | 0 | 2 | 2 | 1 | 2 |
|  | Lower | -38 | -49 | -58 | -57 | -57 | -56 | -58 | -62 | -61 | -66 | -62 | -60 | -61 | -58 |
|  | Upper | 43 | 46 | 55 | 54 | 56 | 56 | 57 | 57 | 56 | 53 | 61 | 62 | 58 | 56 |
| North Macedonia | real | 1 | 2 | 0 | 3 | 5 | 3 | 6 | 8 | 3 | 3 | 5 | 4 | 2 | 4 |
|  | forecast | 5 | 7 | 6 | 6 | 5 | 3 | 5 | 6 | 7 | 8 | 8 | 6 | 4 | 5 |
|  | Lower | 1 | 3 | 2 | 2 | 1 | 0 | 1 | 2 | 3 | 4 | 4 | 2 | 0 | 1 |
|  | Upper | 9 | 11 | 10 | 10 | 8 | 7 | 8 | 11 | 11 | 12 | 12 | 10 | 8 | 9 |
| Norway | real | 0 | 0 | 0 | 0 | 0 | 0 | 0 | 0 | 0 | 1 | 0 | 0 | 0 | 0 |
|  | forecast | 0 | 0 | 0 | 0 | 0 | 0 | 1 | 1 | 1 | 1 | 1 | 1 | 0 | 1 |
|  | Lower | -4 | -4 | -4 | -4 | -4 | -4 | -4 | -4 | -4 | -4 | -4 | -4 | -4 | -4 |
|  | Upper | 4 | 5 | 5 | 5 | 5 | 5 | 5 | 5 | 5 | 5 | 5 | 5 | 5 | 5 |
| Poland | real | 6 | 19 | 20 | 14 | 8 | 13 | 7 | 3 | 12 | 11 | 12 | 10 | 13 | 6 |
|  | forecast | 3 | 6 | 7 | 8 | 8 | 8 | 8 | 8 | 8 | 8 | 8 | 8 | 9 | 9 |
|  | Lower | -8 | -7 | -8 | -7 | -5 | -4 | -5 | -6 | -6 | -7 | -7 | -6 | -5 | -4 |
|  | Upper | 12 | 18 | 21 | 21 | 22 | 23 | 22 | 21 | 22 | 22 | 22 | 22 | 24 | 24 |
| Portugal | real | 2 | 3 | 2 | 4 | 5 | 2 | 3 | 3 | 3 | 3 | 3 | 5 | 7 | 4 |
|  | forecast | 5 | 7 | 8 | 10 | 10 | 9 | 9 | 11 | 12 | 12 | 14 | 13 | 12 | 11 |
|  | Lower | -2 | -2 | -1 | 0 | 0 | -2 | -1 | 0 | 0 | 1 | 2 | 2 | 2 | 0 |
|  | Upper | 13 | 16 | 18 | 20 | 20 | 20 | 20 | 22 | 25 | 24 | 25 | 23 | 23 | 22 |
| Republic of Moldova | real | 13 | 16 | 12 | 11 | 16 | 0 | 11 | 13 | 9 | 10 | 8 | 3 | 6 | 6 |
|  | forecast | 6 | 6 | 7 | 6 | 4 | 3 | 5 | 7 | 5 | 6 | 7 | 5 | 3 | 6 |
|  | Lower | 0 | 0 | 1 | 0 | -2 | -3 | 0 | 2 | -1 | -1 | 0 | -1 | -2 | 0 |
|  | Upper | 11 | 12 | 12 | 12 | 10 | 9 | 11 | 13 | 11 | 12 | 13 | 11 | 9 | 11 |
| Romania | real | 43 | 60 | 40 | 44 | 47 | 38 | 43 | 33 | 41 | 51 | 47 | 35 | 27 | 36 |
|  | forecast | 29 | 23 | 21 | 19 | 18 | 17 | 15 | 16 | 18 | 20 | 21 | 23 | 21 | 19 |
|  | Lower | 18 | 8 | 7 | 5 | 5 | 1 | 1 | 2 | 4 | 7 | 6 | 8 | 6 | 6 |
|  | Upper | 41 | 35 | 35 | 34 | 33 | 31 | 29 | 31 | 33 | 35 | 36 | 37 | 34 | 33 |
| Russian Federation | real | 123 | 115 | 114 | 121 | 110 | 61 | 51 | 122 | 142 | 128 | 102 | 119 | 94 | 57 |
|  | forecast | 91 | 97 | 102 | 105 | 107 | 109 | 109 | 109 | 110 | 111 | 111 | 111 | 111 | 110 |
|  | Lower | 45 | 33 | 39 | 41 | 34 | 43 | 40 | 39 | 41 | 35 | 40 | 29 | 38 | 36 |
|  | Upper | 140 | 164 | 171 | 176 | 176 | 183 | 174 | 176 | 181 | 184 | 177 | 174 | 176 | 173 |
| Serbia | real | 2 | 2 | 1 | 2 | 3 | 2 | 1 | 1 | 2 | 1 | 1 | 1 | 1 | 2 |
|  | forecast | 3 | 4 | 6 | 6 | 7 | 7 | 7 | 7 | 7 | 7 | 8 | 8 | 7 | 7 |
|  | Lower | 0 | 1 | 2 | 3 | 3 | 3 | 3 | 3 | 4 | 3 | 4 | 4 | 4 | 3 |
|  | Upper | 6 | 7 | 9 | 10 | 11 | 11 | 11 | 10 | 11 | 11 | 11 | 12 | 11 | 11 |
| Slovakia | real | 0 | 0 | 4 | 0 | 0 | 0 | 0 | 0 | 0 | 0 | 0 | 1 | 0 | 0 |
|  | forecast | 0 | 0 | 0 | 0 | 0 | 0 | 0 | 0 | 0 | 0 | 0 | 0 | 0 | 0 |
|  | Lower | -1 | -1 | -1 | -1 | -1 | -1 | -1 | -1 | -1 | -1 | -1 | -1 | -1 | -1 |
|  | Upper | 1 | 1 | 1 | 1 | 1 | 1 | 1 | 1 | 1 | 1 | 1 | 1 | 1 | 1 |
| Slovenia | real | 0 | 1 | 0 | 0 | 1 | 0 | 0 | 0 | 0 | 0 | 0 | 0 | 0 | 0 |
|  | forecast | 0 | 1 | 1 | 1 | 1 | 1 | 1 | 1 | 1 | 1 | 1 | 1 | 1 | 1 |
|  | Lower | -2 | -1 | -1 | -1 | -1 | -1 | -1 | -1 | -1 | -1 | -1 | -1 | -1 | -2 |
|  | Upper | 2 | 3 | 2 | 3 | 3 | 3 | 3 | 3 | 3 | 3 | 3 | 3 | 3 | 3 |
| Spain | real | 58 | 42 | 40 | 184 | 82 | 83 | 68 | 78 | 34 | 71 | 48 | 94 | 101 | 98 |
|  | forecast | 82 | 81 | 80 | 79 | 78 | 78 | 77 | 76 | 75 | 76 | 76 | 76 | 76 | 75 |
|  | Lower | -42 | -105 | -136 | -153 | -168 | -224 | -248 | -241 | -256 | -256 | -258 | -247 | -281 | -270 |
|  | Upper | 224 | 264 | 283 | 305 | 324 | 331 | 357 | 385 | 365 | 381 | 424 | 432 | 432 | 428 |
| Sweden | real | 2 | 3 | 2 | 2 | 0 | 0 | 3 | 1 | 1 | 2 | 2 | 4 | 1 | 2 |
|  | forecast | 4 | 4 | 5 | 5 | 6 | 6 | 7 | 7 | 7 | 8 | 8 | 9 | 9 | 9 |
|  | Lower | -10 | -13 | -16 | -18 | -19 | -23 | -24 | -27 | -28 | -26 | -30 | -29 | -29 | -31 |
|  | Upper | 16 | 21 | 26 | 30 | 32 | 37 | 40 | 40 | 42 | 45 | 47 | 48 | 50 | 50 |
| Switzerland | real | 1 | 1 | 3 | 1 | 2 | 2 | 3 | 1 | 1 | 7 | 0 | 4 | 4 | 1 |
|  | forecast | 1 | 2 | 2 | 2 | 2 | 1 | 1 | 2 | 2 | 2 | 2 | 2 | 2 | 2 |
|  | Lower | -6 | -9 | -11 | -11 | -12 | -12 | -12 | -12 | -12 | -11 | -12 | -13 | -13 | -13 |
|  | Upper | 9 | 11 | 13 | 16 | 16 | 16 | 17 | 18 | 18 | 17 | 19 | 18 | 18 | 18 |
| The United Kingdom | real | 11 | 18 | 11 | 15 | 9 | 15 | 14 | 13 | 24 | 16 | 14 | 16 | 19 | 23 |
|  | forecast | 11 | 12 | 14 | 16 | 17 | 16 | 17 | 19 | 20 | 22 | 25 | 25 | 25 | 23 |
|  | Lower | -48 | -68 | -91 | -111 | -120 | -133 | -140 | -143 | -143 | -142 | -138 | -146 | -155 | -170 |
|  | Upper | 78 | 103 | 128 | 143 | 153 | 164 | 169 | 176 | 190 | 187 | 202 | 213 | 215 | 222 |
| Turkey | real | 44 | 47 | 45 | 49 | 53 | 56 | 53 | 57 | 52 | 55 | 58 | 56 | 48 | 57 |
|  | forecast | 42 | 42 | 41 | 41 | 41 | 41 | 41 | 40 | 40 | 40 | 40 | 40 | 39 | 39 |
|  | Lower | 34 | 31 | 28 | 26 | 24 | 23 | 20 | 19 | 18 | 19 | 17 | 15 | 15 | 13 |
|  | Upper | 50 | 54 | 54 | 57 | 58 | 59 | 61 | 62 | 62 | 63 | 65 | 66 | 68 | 67 |
| Ukraine | real | 15 | 12 | 27 | 16 | 22 | 13 | 21 | 24 | 21 | 18 | 27 | 11 | 15 | 14 |
|  | forecast | 21 | 19 | 18 | 18 | 17 | 14 | 13 | 15 | 15 | 16 | 16 | 15 | 14 | 13 |
|  | Lower | 12 | 10 | 8 | 7 | 7 | 5 | 3 | 5 | 6 | 5 | 6 | 4 | 3 | 4 |
|  | Upper | 30 | 29 | 28 | 27 | 28 | 25 | 23 | 24 | 26 | 25 | 26 | 24 | 24 | 24 |
