# Supplementary material for: Application of distributed lag models and spatial analysis for comparing the performance of the COVID-19 control decisions in European countries
Source: Sci Rep. 2023 Oct 14;13:17466. doi: 10.1038/s41598-023-44830-z (PMC10576777; doi:10.1038/s41598-023-44830-z)
Supplement: Supplementary file 4 — Supplementary Information 4. [file 41598_2023_44830_MOESM4_ESM.docx]

rm(list=ls())

library(dLagM)

###################### coparison three model ##########################

########################## AIC ######################################

data <- read.csv("data.csv")

name.country=unique(data$Country)

aic <- matrix(NA, nrow = length(name.country), ncol = 3,

dimnames = list(name.country, c("DLM", "PolyDLM", "KoyckDLM")))

for (i in 1:length(name.country)) {

data4 <- subset(data, Country == name.country[i])

dlmFit1 <- dlm(New_deaths~New_cases,data = data4 , q = 14)

aic[name.country[i], "DLM"] <- AIC(dlmFit1)

x <- data4$New_cases

y <- data4$New_deaths

polymodel=polyDlm(x,y,q = 14,k=2)

aic[name.country[i], "PolyDLM"] <- AIC(polymodel)

KoyckFit <- koyckDlm(x,y)

aic[name.country[i], "KoyckDLM"] <- AIC(KoyckFit)

}

aic

############################################# MSE ###################

mse <- matrix(NA, nrow = length(name.country), ncol = 3,

dimnames = list(name.country, c("DLM", "PolyDLM", "KoyckDLM")))

for (i in 1:length(name.country)) {

data4 <- subset(data, Country == name.country[i])

dlmFit1 <- dlm(New_deaths~New_cases,data = data4 , q = 14)

x <- data4$New_cases

y <- data4$New_deaths

polymodel=polyDlm(x,y,q = 14,k=2)

KoyckFit <- koyckDlm(x,y)

mse[name.country[i], "DLM"] <- GoF(dlmFit1,polymodel,KoyckFit)$ MSE[1]

mse[name.country[i], "PolyDLM"] <- GoF(dlmFit1,polymodel,KoyckFit)$ MSE[2]

mse[name.country[i], "KoyckDLM"] <- GoF(dlmFit1,polymodel,KoyckFit)$ MSE[3]

}

mse

####################################################### coeff #########

coef.DLM<- matrix(NA, nrow = 16, ncol = length(name.country),

dimnames = list(c("intercept",paste0("Lag", 0:14)), name.country))

coef.ployDLM<- matrix(NA, nrow = 15, ncol = length(name.country),

dimnames = list(c(paste0("Lag", 0:14)), name.country))

lambda.koyckDLM <- matrix(NA, nrow =2 , ncol = length(name.country),

dimnames = list( c("lambda","beta0"),name.country))

for (i in 1:length(name.country)) {

data4 <- subset(data, Country == name.country[i])

dlmFit1 <- dlm(New_deaths~New_cases,data = data4 , q = 14)

c=matrix(coef(dlmFit1),16,1,byrow=T)

coef.DLM[,i]=c

x <- data4$New_cases

y <- data4$New_deaths

polymodel=polyDlm(x,y,q = 14,k=2)

b=polymodel$beta

coef.ployDLM[,i]<- b$beta

KoyckFit <- koyckDlm(x,y)

a=matrix(coef( KoyckFit))

lambda.koyckDLM["lambda",name.country[i]] <-a[2,1]

lambda.koyckDLM["beta0",name.country[i]] <-a[3,1]

}

coef.DLM=coef.DLM[-1,];coef.DLM

coef.ployDLM

lambda.koyckDLM

############# mean delay and long run effect################

mean.delay=matrix(NA,39,3,dimnames = list(name.country, c("DLM", "PolyDLM", "KoyckDLM")))

long.run.effect=matrix(NA,39,3,dimnames = list(name.country, c("DLM", "PolyDLM", "KoyckDLM")))

for (i in 1:length(name.country)) {

j=c(1:15)

mean.delay[name.country[i],"DLM"]=sum((j-1)*coef.DLM[j,i])/sum(coef.DLM[,i])

long.run.effect[name.country[i],"DLM"]=sum(coef.DLM[,i])

mean.delay[name.country[i],"PolyDLM"]=sum(j*coef.ployDLM[j,i])/sum(coef.ployDLM[,i])

long.run.effect[name.country[i],"PolyDLM"]=sum(coef.ployDLM[,i])

mean.delay[name.country[i],"KoyckDLM"]=lambda.koyckDLM[1,i]/(1-lambda.koyckDLM[1,i])

long.run.effect[name.country[i],"KoyckDLM"]=lambda.koyckDLM[2,i]/(1-lambda.koyckDLM[1,i])

}

mean.delay

long.run.effect

############################forecast

xdata <- read.csv("observed.csv")

name.country <- unique(data$Country)

# Convert "Country" variable to factor with desired levels

data$Country <- factor(data$Country, levels = name.country)

xdata$Country <- factor(xdata$Country, levels = name.country)

forecast.DLM <- matrix(NA, nrow = 14, ncol = length(name.country)*3,

dimnames = list(paste0("day", 1:14), rep(name.country,each = 3)))

for (i in 8:length(name.country)) {

data4 <- subset(data, Country == name.country[i])

dlmFit1 <- koyckDlm(data4$New_cases,data4$New_deaths)

xdata1 <- subset(xdata, Country == name.country[i])

x <- as.numeric(unlist(xdata1$New_cases))

forecast.DLM[, (3*i-2):(3*i)] <-round( as.matrix(forecast(dlmFit1, x = x, h = 14, interval = TRUE)$forecasts))

}

forecast.DLM
